# Case report: Clinical success targeting BRAF-mutated, hormone receptor positive, HER2- negative advanced breast cancer patient with BRAF-inhibitor plus MEK- inhibitor

**DOI:** 10.3389/fonc.2022.997346

**Published:** 2022-12-02

**Authors:** Alfonso López de Sá, Alicia de Luna, Mónica Antoñanzas, Vanesa García-Barberán, Fernando Moreno-Anton, Jose A. García-Sáenz

**Affiliations:** ^1^ Servicio de Oncología Médica, Instituto de Investigación Sanitaria Hospital Clinico San Carlos (IdISSC), Madrid, Spain; ^2^ Laboratorio de Oncología Molecular, Instituto de Investigación Sanitaria Hospital Clinico San Carlos (IdISSC), Madrid, Spain

**Keywords:** BRAF, ESCAT, HR+, advanced breast cancer, trametinib, dabrafenib, case report

## Abstract

Background: Hormone receptor-positive, human epidermal growth factor 2-negative advanced breast cancer patients have had a wide range of therapeutical options since the incorporation of targeted therapies alongside classic chemotherapy. However, because of their disease, virtually all patients will eventually experience disease progression that might compromise their lives. Thriving investigation regarding molecular therapies has provided clinicians with new options for the treatment of many cancer patients. Dabrafenib and trametinib combination has proven useful in treating malignant melanoma patients harboring a BRAF V600E mutation, improving progression-free survival and overall survival, and it has been tested in other tumors. Here we report the case of a metastatic breast cancer patient harboring a BRAF V600E mutation that achieved complete response with dabrafenib and trametinib combination.

## Introduction

Endocrine therapies (ET) plus targeted-therapies including CDK4/6-inhibitors, PI3K/AKT/mTOR inhibitors have dramatically improved the long-term outcomes in patients with hormone receptor-positive (HR+), human epidermal growth factor 2- negative (HER2–) advanced breast cancer (1) (BC). Despite these improvements, almost all the HR+/HER2- metastatic BC patients will experience disease progression. Hence, new potential sequential therapies deserve to be further investigated.

The increasing knowledge of the biology of the disease has allowed clinicians to identify new actionable targets, in order to prevent or reverse resistance to previous therapies (2). The European Society of Medical Oncology (ESMO) Scale for Clinical Actionability of molecular Targets (ESCAT) (**Table 1**) provides a framework to assign DNA alterations into tiers that reflect their clinical utility when selecting patients for treatment with targeted therapies (3).

**Table 1:** ESMO Scale for Clinical Actionability of molecular Targets (ESCAT).

ESCAT evidence tier	Level of evidence A	Level of evidence B	Level of evidence C	Clinical implications
I: Alteration-drug match is associated with improved outcome in clinical trials	Prospective, randomized clinical trials show the alteration drug match in a specific tumor type results in a clinically meaningful improvement of a survival end point	Prospective, non-randomized clinical trials show that the alteration-drug match in a specific tumor type, results in clinically meaningful benefit as defined by ESMO MCBS 1.1	Clinical trials across tumor types or basket clinical trials show clinical benefit associated with the alteration- drug match, with similar benefit observedacross tumor types	Access to the treatment should be considered standard of care
II: Alteration-drug match is associated with antitumor activity, but magnitude of benefit is unknown	Retrospective studies show patients with the specific alteration in a specific tumor type experience clinically meaningful benefit with matched drug compared with alteration- negative patients	Prospective clinical trial(s) show the alteration- drug match in a specific tumor type results in increased responsiveness when treated with a matched drug, however, no data currently available on survival end points	NA	Treatment to be considered ‘preferable’ in the context of evidence collection either as a prospective registry or as a prospective clinical trial
III: Alteration-drug match suspected to improve outcome based on clinical trial data in other tumor type(s) or with similar molecular alteration	Clinical benefit demonstrated in patients with the specific alteration (as tiers I and II above) but in a different tumor type.Limited/absence of clinical evidence available for the patient- specific cancer type or broadly across cancer type	An alteration that has a similar predicted functional impact as an already studied tier I abnormality in the same gene or pathway, but does not have associated supportive clinical data	NA	Clinical trials to be discussed with patients
IV: Preclinical evidence of actionability	Evidence that the alteration or a functionally similar alteration influences drug sensitivity in preclinic *in vitro* or *in vivo* models	Actionability predicted in silico	NA	Treatment should ‘only be considered’ in the context of early clinical trials. Lack of clinical data should be stressed to patients
V: Alteration-drug match is associated with objective response, but without clinicallymeaningful benefit	Prospective studies show that targeted therapy is associated with objective responses, but this does not lead to improved outcome			Treatment should ‘only be considered’ in the context of early clinical trials. Lack of clinical data should be stressed to patients. Clinical trials assessing drug combinationstrategies could be considered.
X: Lack of evidence for actionability	No evidence that the genomic alteration is therapeutically actionable			The finding should not be taken into account for clinical decision

To date, the genomic alteration of MAPK signaling pathway and the potential actionability in BC is allocated in the TIER III ESCAT classification, meaning that suspected clinical benefit targeting this alteration is based on clinical trial data in other tumor type or in those with similar molecular alteration.

The MAPK signaling pathway communicates a signal from a receptor on the surface of the cell to the nucleus and is compounded by RAS-RAF-MEK-ERK (4, 5) and there are several down-regulator controls that limit physiologic activation of MAPK signaling. The frequency of genomic alterations in the MAPK pathway decreases in incidence as one moves further downstream in the pathway: across human tumors, RAS mutations occur in 22%, BRAF in 7%, MEK in <1% of cases and ERK mutation are exceptionally rare (6).

BRAF alterations are present in several other tumor histologies, including cutaneous melanomas (50%), thyroid cancer (20–50%), colorectal cancer (10%), non-small-cell lung cancer (NSCLC) (2–4%), and hairy cell leukemia (>90%) (7).

The point mutation substitution of BRAF exon-15 (V600E) is the most common mutation across all tumor types and the class of BRAF mutation has clinical implications, since BRAF inhibitors have been mainly tested against class 1 monomer-type mutations (V600 mutations) (8).

BRAF alterations are extremely rare in BC, but they are of potential therapeutic interest because they can be targeted with kinase inhibitors. According to the AACR Project GENIE Consortium database, BRAF is altered in 1.38% of breast carcinoma patients with BRAF V600E present in only 0.11% (9, 10).

Albanell et al. analyzed 7850 BC tumors with a total of 83 (1.1%) BRAF alterations identified. BRAF alterations that may lead to aberrant MAPK signaling included amplifications (51.8%), V600E substitution (15.7%), other missense substitution (25.2%), and fusions (6.0%). Of the cases harboring altered BRAF, 38.6% were triple negative breast cancer, 21.7% HR +/HER2-, 2.4% HR-/HER2 +, 2.4% HR +/HER2 +, and 30.1% status unknown (11).

Current targeted therapies for patients harboring a BRAF mutation include a combination of BRAF and MEK inhibitors, which were originally developed to treat metastasic melanoma. There are scarce but promising literature reports of success using these agents against breast cancer patients (12–14).

## Case report

We present the case of a 41-year-old premenopausal woman not harboring pathogenic germline BRCA mutation who was diagnosed in 2009, at the age of 29, with an early stage, HR+/HER2- BC. She underwent radical surgery, taxane-anthracycline based adjuvant chemotherapy and adjuvant ET with ovarian function suppression (OFS) and tamoxifen for 5 years.

In July 2015, less than 1 year after finishing endocrine treatment, a histological HR+/HER2- proven bone relapse was diagnosed. Endocrine first palliative line treatment with OFS and an aromatase inhibitor (AI) was started, achieving a 26-month period of disease control and then, new bone and liver metastases were observed. Second ET line with Palbociclib, OFS and Fulvestrant was started obtaining a clinical benefit and a 9 month progression free survival (PFS). PIK3CA status at this time revealed a PIK3CA-wild type bone recurrence, so she started a third ET line with exemestane plus everolimus and OFS for 11 months. A subsequent line of abemaciclib, tamoxifen and OFS was started, obtaining 13 months of disease control.

A comprehensive genomic profiling, using the Foundation Medicine^®^ platform was performed in order to look for actionable targets, and a BRAF p.V600E mutation was found. The patient refused to undertake chemotherapy due to potential side effects and was eager to explore other options; so, once discussed in our institution molecular board and adequately explained to the patient the current evidence of tyrosine kinase therapy in BC, she agreed to start an off-label treatment targeting BRAF pathway. The combination of dabrafenib 150mg every 12 hours and trametinib 2mg daily was initiated in March, 2021.

The treatment related adverse events (trAEs) were similar to those reported in previous studies: with grade 2 nausea and pyrexia as the main toxicities after 10 days of treatment, which lead to temporary treatment discontinuation. As grade 2 pyrexia recurred and persisted after restarting therapy, the doses of both drugs were lowered to dabrafenib 100 mg every 12 hours and trametinib 1.5 mg daily, with no new trAEs in the following months.

**Figure 1:**
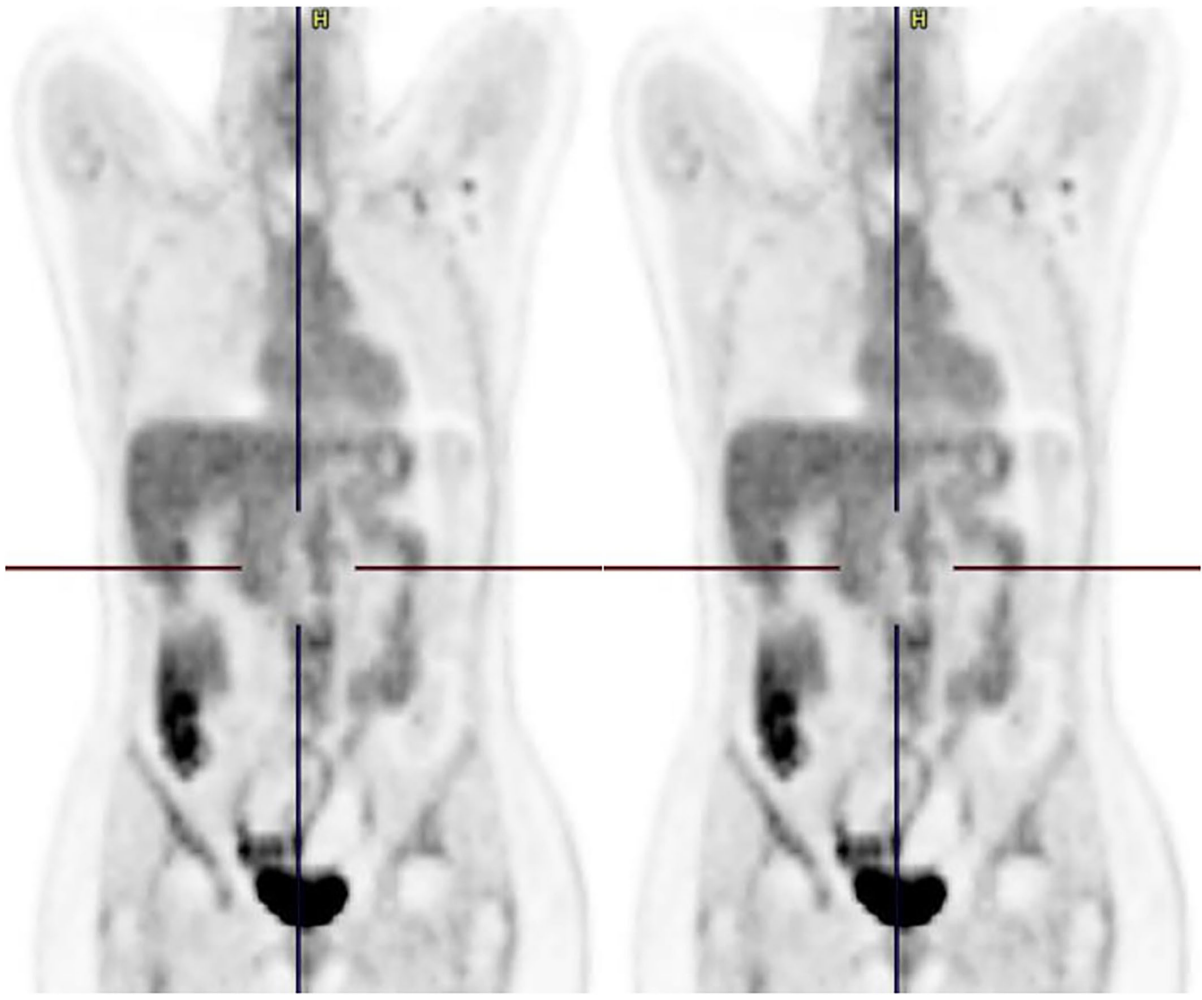
Coronal reformatted PET image before and after treatment initiation. Figure 1 (left) shows two hypermetabolic liver lesions. Figure 2 (right) shows complete response of both lesions.

**Figure 2:**
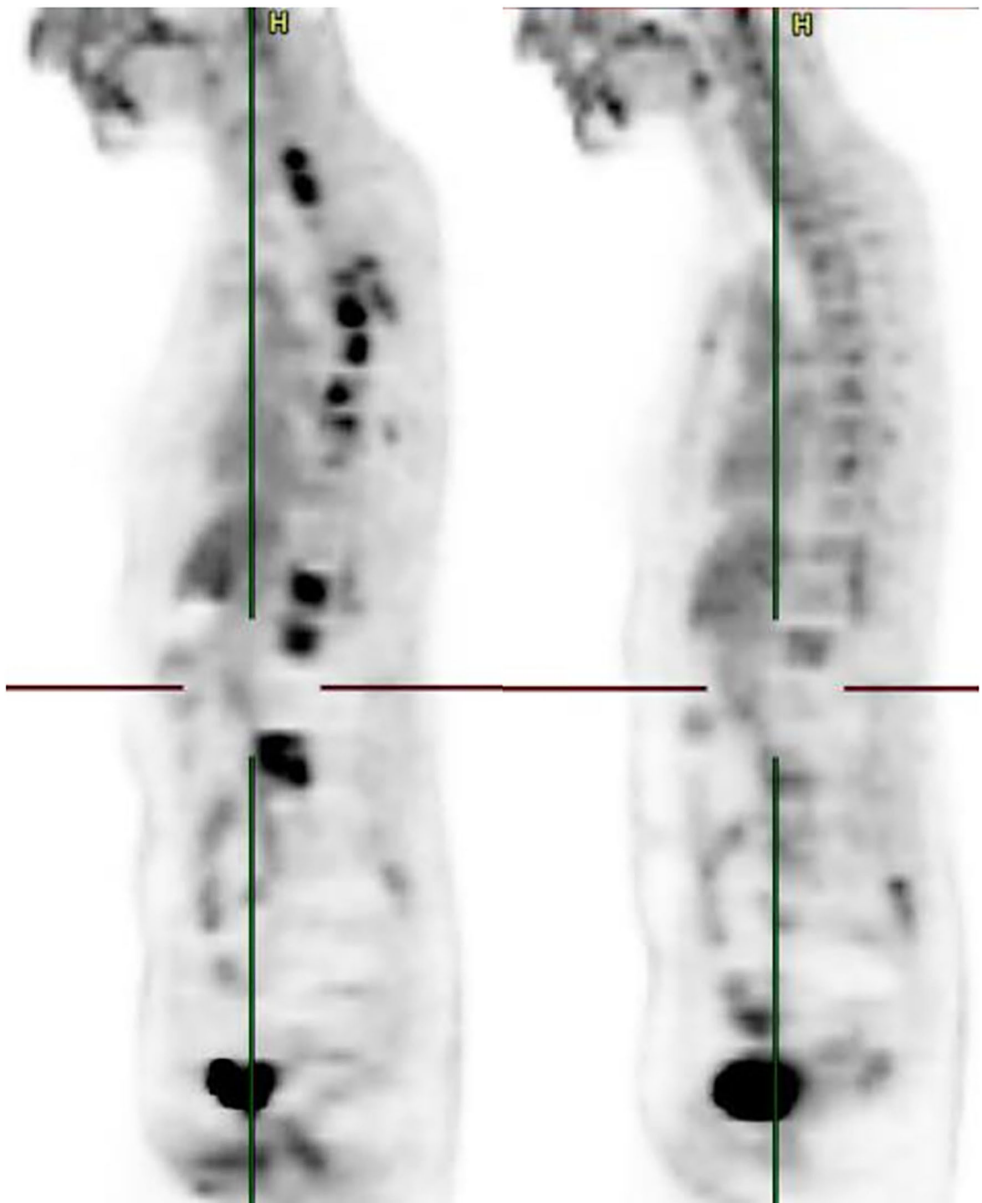
Sagittal reformatted PET image before and after treatment initiation. Multiple foci of intense bone uptake are shown in figure 3 (left). Figure 4 (right) shows metabolic response of every lesion.

First imaging reevaluation was performed in June, 2021 using a CT-PET scan that showed hepatic and bone complete response (**Figures 1**, **2**). The patient experienced clinical benefit and no further significant toxicity, maintaining her quality of life, until December 2021, when bone progression was documented. Therefore, a PFS of 9 months was achieved with excellent performance status during treatment.

**Figure 3:**
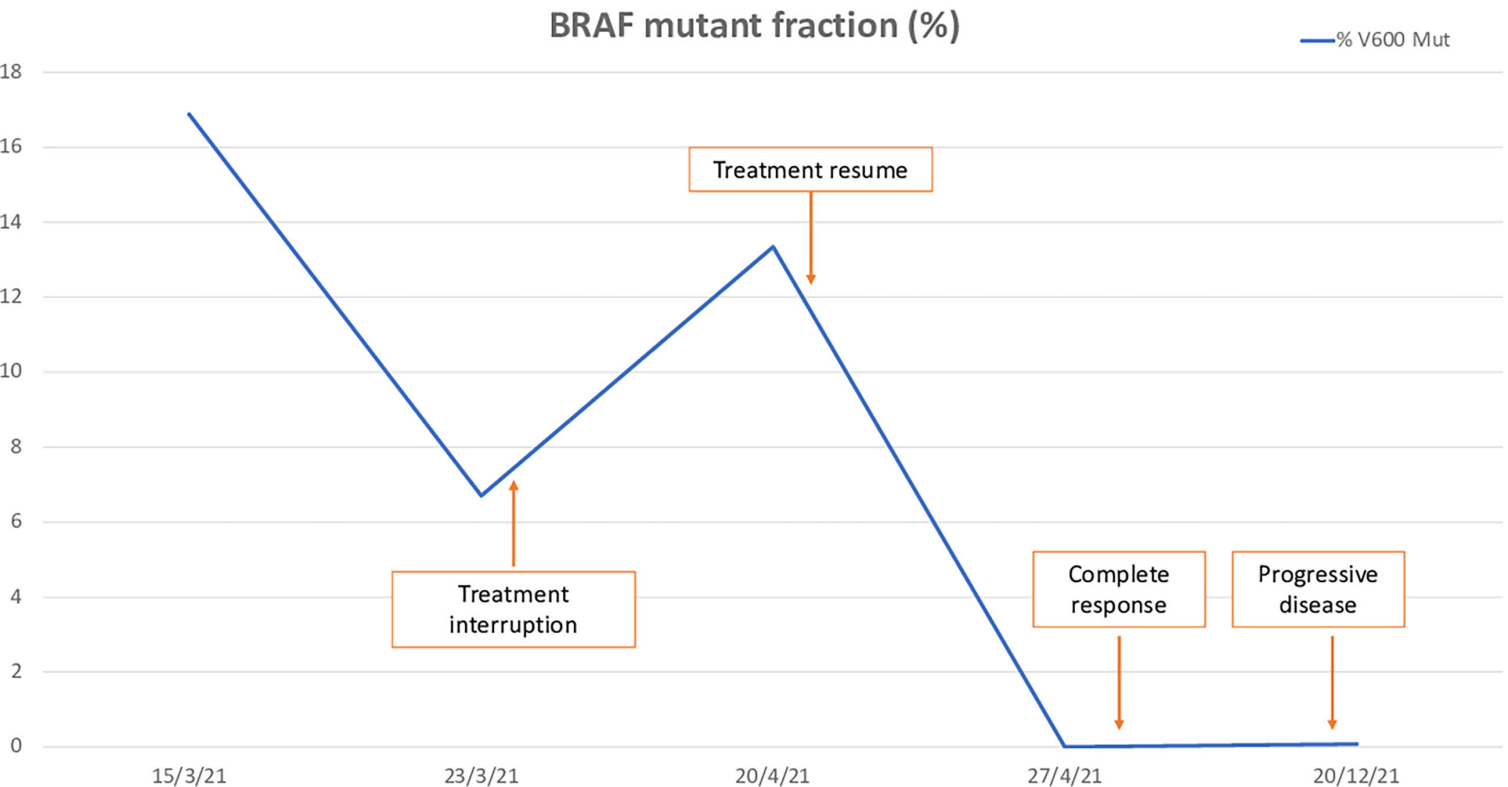
Mutant allele frequency of BRAF V600E mutation in plasma during treatment.

Since the beginning of treatment, the patient has been monitored by liquid biopsy, studying blood-circulating DNA harboring BRAF V600E mutation (ctBRAF). Blood was collected in EDTA tubes at 4 initiating time points, and plasma was processed before one hour after extraction. BRAF V600E mutation in plasma was evaluated by Idylla^®^ ctBRAF Mutation assay (Biocartis^®^) (**Figure 3**). Mutant allele frequency (MAF) of 16.9% was detected before start the dabrafenib/trametinib treatment. In a second time point (+8 days of treatment), mutation was reduced to 6.7%, which could suggest a benefit of this treatment. During temporary treatment discontinuation by trAEs, the liquid biopsy showed an increase of mutation frequency (MAF of 13.3%). Lastly, mutation was undetectable in plasma after 43 days after the beginning of the treatment, and maintained until disease progression was observed and a slight elevation of MAF to 0.1% was documented in December 2021.

## Discussion

The combination of BRAF and MEK inhibitors is actually the standard of care for patients harboring BRAF V600 activating mutation melanoma, either dabrafenib plus trametinib, vemurafenib and cobimetinib or encorafenib plus binimetinib.

Furthermore, dabrafenib plus trametinib combination has FDA approval for anaplastic thyroid cancer (ATC), metastasic NSCLC, and advanced biliary tract cancers, and encorafenib plus binimetinib plus cetuximab has FDA and EMA approval for second line colorectal carcinoma.

Regarding other tumor histologies, the efficacy of these combinations is limited to some basket trials in BRAF V600E patients, and few case reports are published. In the single-arm phase II study VE-BASKET Diamond et al. observed preliminary activity of vemurafenib monotherapy in 172 patients with solid tumors including NSCLC, histiocytic neoplasms, glioma, ATC and GI tumors with ORR of 32.6% (95%CI: 25.6-40.1%) (15).

The ROAR phase II study was designed for 9 rare BRAF-mutant cancers to assess the safety and efficacy of dabrafenib and trametinib therapy with various results published leading to the previous mentioned drug approvals (16).

Neither of these basket trials have included breast cancer patients, and to our knowledge, there is no other clinical trial in progress that aims to include these patients.

A recently published case in *Case Reports in Oncological Medicine* is, as far as we know, the first reported case of the off-label combination of BRAF and MEK inhibitors in metastatic BC. A patient with a locally advanced metaplastic breast carcinoma harboring a BRAF V600E mutation rapidly progressing and refractory to conventional therapy received dabrafenib and trametinib with initial response rate and an important tumor shrinkage within 2 first weeks of treatment. Overall, the duration of response was 7 weeks (7). A second reported case regarding a breast cancer patient treated with a BRAF has recently been published (8). Pircher and colleagues report the case of a 38-year-old woman with a BRAF mutated metastatic triple-negative breast cancer that had progressed to two prior therapies and vemurafenib treatment was initiated. The patient experienced a partial response in the first performed CT scan after 3 months of treatment and maintains it after 19 months of follow-up at the time of the report.

## Conclusion

Although metastatic HR+/HER2- breast cancer patients have nowadays many interesting therapeutic options, clinicians eventually find themselves struggling with hormone resistance and the need to use traditional chemotherapy that, in many cases, leads to suboptimal results and significant toxicities. Next generation sequencing offers thorough information regarding the molecular landscape of the tumor that may be of use when exploring new options for breast cancer patients.

Albeit rare, BRAF V600E mutations may be of potential therapeutic interest, since good results have been obtained in other tumor types, as stated above. Close monitoring with ctBRAF may lead to better clinical control (9). Further investigation is needed to explore its efficacy in breast cancer patients.

## Data availability statement

The original contributions presented in the study are included in the article/supplementary material. Further inquiries can be directed to the corresponding author.

## Ethics statement

Written informed consent was obtained from the individual(s) for the publication of any potentially identifiable images or data included in this article.

## Author contributions

JG-S, FA, AL and AS participated in the patient’s medical treatment. MA provided expertise regarding BRAF-mutated patients. VG-B performed molecular techniques necessary for the follow-up. AS and JG-S wrote the first manuscript and all authors contributed to reviewing it. All authors contributed to the article and approved the submitted version.

## Conflict of interest

The authors declare that the research was conducted in the absence of any commercial or financial relationships that could be construed as a potential conflict of interest.

## Publisher’s note

All claims expressed in this article are solely those of the authors and do not necessarily represent those of their affiliated organizations, or those of the publisher, the editors and the reviewers. Any product that may be evaluated in this article, or claim that may be made by its manufacturer, is not guaranteed or endorsed by the publisher.
